# Remimazolam besylate versus propofol for deep sedation in critically ill patients: a randomized pilot study

**DOI:** 10.1186/s13054-023-04760-8

**Published:** 2023-12-04

**Authors:** Yun Tang, Xuehui Gao, Jiqian Xu, Lehao Ren, Hong Qi, Ruiting Li, Huaqing Shu, Xiaojing Zou, Shiying Yuan, Xiaobo Yang, You Shang

**Affiliations:** Department of Critical Care Medicine, Union Hospital, Tongji Medical College, Huazhong University of Science and Technology, Wuhan, China

**Keywords:** Remimazolam, Propofol, Sedation, Critical care

## Abstract

**Objective:**

To compare the efficacy and safety of remimazolam besylate and propofol for deep sedation in critically ill patients.

**Methods:**

In this single-center, prospective, randomized, controlled pilot study, patients in the intensive care unit (ICU) requiring deep sedation were randomized to receive remimazolam besylate or propofol intravenously. Deep sedation was defined as a Richmond Agitation and Sedation Scale (RASS) score of − 4 or − 5. Sedation depth was monitored using RASS and Narcotrend Index (NI). The primary outcome was the percentage of time within the target sedation range without rescue sedation. The secondary outcomes included ventilator-free hours within 7 days, successful extubation, length of ICU stay, and 28-day mortality. Adverse events during the interventional period were also recorded.

**Results:**

Thirty patients were assigned to each group. The median (IQR) RASS score was − 5.0 (− 5.0, − 4.0), and the median (IQR) NI value was 29.0 (21.0, 37.0) during the intervention period. Target RASS was reached a median of 100% of the sedation time in the two groups. No significant differences were observed in ventilator-free hours within 7 days, successful extubation, length of ICU stay, or 28-day mortality among groups. Hypotension occurred in 16 (53.3%) patients of remimazolam group and 18 (60.0%) patients of propofol group (*p* > 0.05). No patient experienced bradycardia.

**Conclusions:**

Remimazolam besylate appears to be an effective and safe agent for short-term deep sedation in critically ill patients. Our findings warrant large sample-sized randomized clinical trials.

**Supplementary Information:**

The online version contains supplementary material available at 10.1186/s13054-023-04760-8.

## Introduction

Sedation is an essential therapy for most mechanically ventilated patients in the intensive care unit (ICU) [1]. The current guideline recommend minimal sedation strategy in adult ICU patients [2], but there are exceptions, such as severe acute respiratory distress syndrome (ARDS) or compromised hemodynamics, where deep sedation is usually required [3, 4].

Midazolam, a benzodiazepine commonly used in mechanically ventilated ICU patients, is associated with a high risk of delirium and prolonged mechanical ventilation [2]. Remimazolam besylate is a new benzodiazepine that has the potential to replace midazolam and propofol with a faster onset and recovery than midazolam and a more stable hemodynamics than propofol [5, 6]. Our previous studies reported that remimazolam besylate was comparable to propofol in maintaining light-to-moderate sedation in ICU patients [7, 8]. However, the research regarding its use for deep sedation lacks. The aim of this pilot study was to preliminarily compare the efficacy and safety of remimazolam besylate and propofol for deep sedation in critically ill patients.

## Methods

### Study design

This single-center, prospective, randomized, controlled study was conducted from September 2022 to May 2023 in Union Hospital, Wuhan, China. The Ethics Committee of our hospital approved this study (2022-0539-01). Written informed consent was obtained from legally representatives. The study was registered (ClinicalTrials.gov, NCT05539521).

### Patients

The inclusion criteria were age between 18 and 80 years, expected to receive mechanical ventilation for longer than 8 h, and the need for intravenous sedative medication for deep sedation, defined as a Richmond Agitation and Sedation Scale (RASS) of − 4 or − 5. The main exclusion criteria were acute severe neurological disorder or coma, systolic blood pressure less than 90 mm Hg after appropriate intravenous volume replacement and vasopressors, and heart rate less than 50 beats/min or second- or third-degree heart block in the absence of a pacemaker (complete list of exclusion criteria in Additional file 1).

### Randomization

Eligible patients were randomized into either group in a 1:1 ratio by opening consecutively numbered, sealed, opaque envelopes with computer-generated allocation. Patients were blinded to allocation, but medical staff were not.

### Intervention

The degree of sedation was measured and recorded every 4 h using RASS. The Narcotrend Index (NI) value was continuously monitored at the same time using Narcotrend-Compact M (MT MonitorTechnik, Germany) and recorded every 4 h.

The remifentanil infusion was started at an initial rate of 6.0 μg/kg/h and titrated to obtain adequate pain control. Patients allocated to the remimazolam group received remimazolam besylate (Yichang Humanwell Pharmaceutical Co., Ltd., China) intravenously at an initial infusion rate of 0.3 mg/kg/h and adjusted (maximum of 3.0 mg/kg/h) to achieve the desired level of sedation. Patients allocated to the propofol group received propofol (Fresenius Kabi China Co., Ltd.) intravenously at an initial infusion rate of 3.0 mg/kg/h and adjusted (maximum of 12.0 mg/kg/h) to achieve the desired level of sedation. If deep sedation was not achieved at the maximum infusion rate, rescue sedation with midazolam was used. The study was continued until one of the following occurred first: 48 h after enrollment, no need for deep sedation, discharge from our ICU, death, or requested cessation by attending physicians or investigators. If a patient still required sedation after stopping the intervention, other sedatives were given at the discretion of attending physicians.

### Outcomes

The primary outcome was the percentage of time within the target sedation range without rescue sedation. The secondary outcomes included ventilator-free hours within 7 days, successful extubation (defined as no reintubation or tracheostomy within 48 h after extubation), length of ICU stay, and 28-day mortality. Patients were considered hospitalized for 28 days if died within 28 days. During the study period, occurrence of hypotension (systolic blood pressure below 80 or diastolic blood pressure below 50) and bradycardia (heart rate below 50) were recorded. Bradycardia was treated with medication to increase heart rate, and hypotension was treated with vasopressors.

### Statistical analysis

This pilot study with no sample size estimation conducted was expected to provide data to calculate sample size for further larger sample-sized trials. Normally distributed variables were reported as mean (standard deviation) and analyzed using the Student’s t test, non-normally distributed variables were reported as median (interquartile range) and analyzed using the Mann–Whitney U test, and categorical variables were reported as number (%) and analyzed using the Chi-square test or the Fisher's exact test. We used Kaplan–Meier plot to present the duration of mechanical ventilation from enrollment to 7 days. Statistical analysis was performed using SPSS 26.0 software (IBM SPSS Statistics, Armonk, NY) and GraphPad Prism 8.2.1 (GraphPad Software, San Diego, CA, USA). A *p* value < 0.05 was considered statistically significant.

## Results

**Fig. 1:**
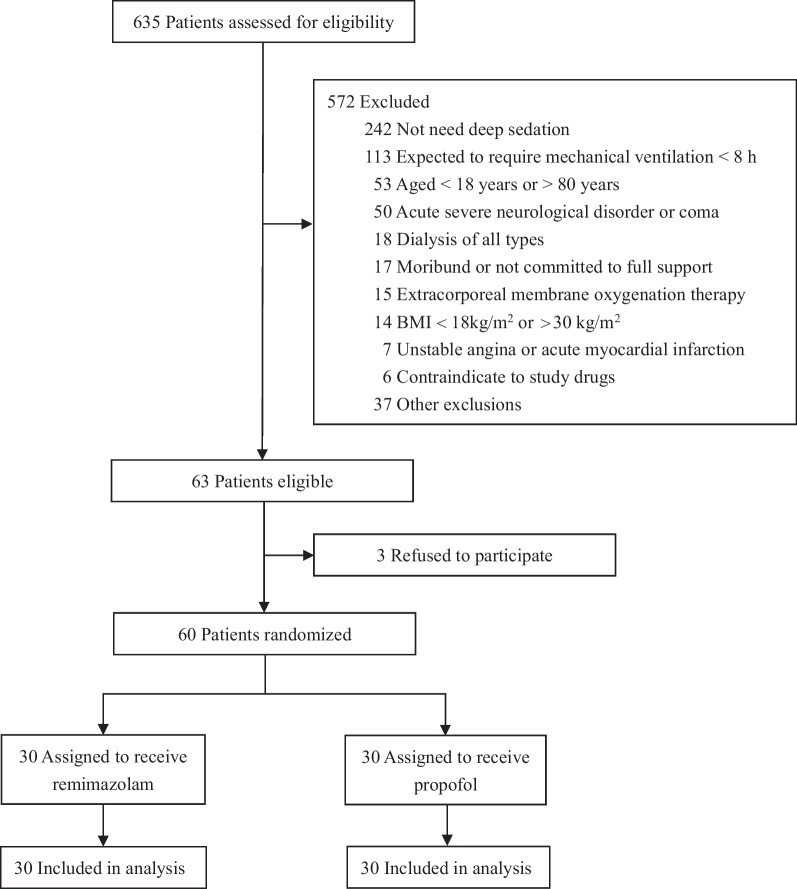
Patient screening, enrollment, and randomization

A total of 635 patients were screened, and 60 patients were randomized (Fig. 1). Their median (IQR) age was 63.0 (55.5–69.0) years, and 41 (68.3%) were male. Patient demographics and baseline characteristics were similar between the two groups (Table 1).

**Table 1:** Demographic and clinical characteristics of the patients at baseline

Characteristic	Remimazolam (n = 30)	Propofol (n = 30)	*p* value
Age, years	62.0 (52.0, 69.5)	64.5 (57.8, 69.3)	0.525
Male	21 (70.0%)	20 (66.7%)	0.781
BMI, kg/m^2^	23.1 ± 2.9	24.1 ± 3.1	0.222
APACHE II score	16.0 (13.8, 18.0)	16.0 (12.0, 17.3)	0.947
SOFA score	7.0 (4.0, 8.0)	7.5 (5.0, 9.0)	0.146
RASS score	− 5.0 (− 5.0, − 4.0)	− 5.0 (− 5.0, − 5.0)	0.139
Narcotrend index	34.3 (9.7)	29.7 (9.3)	0.067
Duration of MV before randomization, h	23.5 (14.0, 52.3)	23.0 (13.0, 46.3)	0.970
Type of admission			
Medical	14 (46.7%)	12 (40.0%)	
Surgical	13 (43.3%)	13 (43.3%)	
Trauma	3 (10.0%)	5 (16.7)	
Reasons for deep sedation			
ARDS	24 (80.0%)	21 (70.0%)	
Sepsis	1 (3.3%)	7 (23.3%)	
Multiple rib fractures	1 (3.3%)	1 (3.3%)	
Hemorrhagic shock	1 (3.3%)	1 (3.3%)	
Patient-ventilator asynchrony	2 (6.7%)	0 (0.0%)	
Heart failure	1 (3.3%)	0 (0.0%)	
Comorbidities			
Hypertension	11 (36.7%)	13 (43.3%)	0.598
Coronary artery disease	6 (20.0%)	5 (16.7%)	0.739
COPD	3 (10.0%)	0 (0.0%)	0.237
Diabetes	7 (23.3%)	4 (13.3%)	0.317
Cancer	5 (16.7%)	6 (20.0%)	0.739
Pre-study sedative			
Remimazolam	12 (40.0%)	9 (30.0%)	0.417
Propofol	5 (16.7%)	6 (20.0%)	0.739
Midazolam	18 (60.0%)	19 (63.3%)	0.791
Dexmedetomidine	2 (6.7%)	2 (6.7%)	1.000
Organ dysfunction			
PaO_2_/FiO_2_ ratio, mmHg	192.5 (147.5, 279.8)	196.5 (119.5, 238.0)	0.657
Vasopressors	15 (50.0%)	20 (66.7%)	0.190
Platelets, 10^9^/L	146.5 (93.0, 236.8)	136.0 (80.0, 184.3)	0.367
Bilirubin, μmol/L	15.6 (11.3, 29.7)	20.0 (17.2, 33.4)	0.246
Creatinine, μmol/L	88.9 (61.1, 122.3)	97.1 (62.6, 148.3)	0.455

Data are number (%), mean (SD), or median (interquartile range)

APACHE II: Acute Physiology and Chronic Health Evaluation II; ARDS: Acute Respiratory Distress Syndrome; BMI: Body Mass Index; COPD: Chronic Obstructive Pulmonary Disease; FiO_2_: Partial pressure of arterial oxygen; MV: Mechanical Ventilation; PaO_2_: Fraction of inspired oxygen; RASS: Richmond Agitation and Sedation Scale; SOFA: Sequential Organ Failure Assessment

**Fig. 2:**
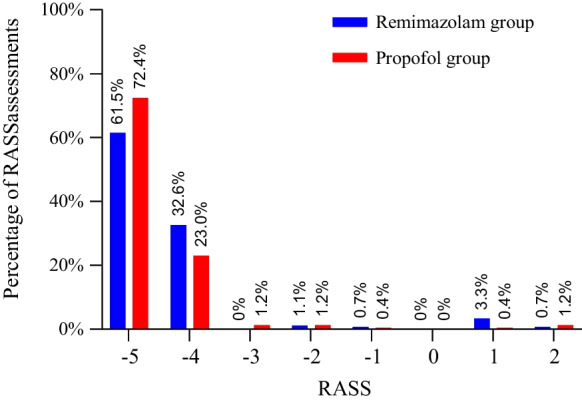
Percentage of Richmond Agitation Sedation Scale (RASS) assessments

The median (IQR) intervention period was 48.0 (21.5, 48.0) hours in the remimazolam group and 28.0 (23.5, 48.0) hours in the propofol group. The median (IQR) infusion rate of remimazolam besylate and propofol were 0.60 (0.45, 1.07) mg/kg/h and 2.53 (1.94, 2.94) mg/kg/h, respectively. Remifentanil infusion rate and the requirement of rescue sedation were similar between groups. The median percentage of time within the target RASS score was 100% in the two groups (Table 2). A total of 273 and 243 RASS assessments were performed during the infusion of remimazolam besylate and propofol, respectively. The target RASS score was achieved in 257 (94.1%) assessments in the remimazolam group and 232 (95.5%) assessments in the propofol group (Fig. 2). The median (IQR) RASS score was -5.0 (-5.0, -4.0), and the median (IQR) NI value was 29.0 (21.0, 37.0) during the intervention period (Additional file 1: Figure S1 and S2). Most of the NI values were in stage D and E (Additional file 1: Figure S3). In most cases, RASS scores and NI values matched well (Additional file 1: Figure S4).

**Table 2:** Intervention, sedation monitoring, and study outcomes

	Remimazolam (n = 30)	Propofol (n = 30)	*P* value
*Intervention and sedation monitoring*			
Study drug treatment			
Duration of infusion, h	48.0 (21.5, 48.0)	28.0 (23.5, 48.0)	0.234
Infusion rate, mg/kg/h	0.60 (0.45, 1.07)	2.53 (1.94, 2.94)	–
Infusion rate of remifentanil, μg/kg/h	6.0 (6.0, 6.3)	6.0 (5.7, 6.1)	0.099
Received rescue sedation	3 (10.0%)	0 (0.0%)	0.237
Received neuromuscular blockers	12 (40.0%)	7 (23.3%)	0.165
Received norepinephrine	21 (70.0%)	24 (80.0%)	0.371
Infusion rate, μg/kg/min	0.06 (0.01, 0.19)	0.07 (0.04, 0.31)	0.148
Prone positioning	12 (40.0%)	7 (23.3%)	0.165
RASS score	− 5.0 (− 5.0, − 4.0)	− 5.0 (− 5.0, − 4.0)	0.717
Narcotrend Index value	28.0 (21.0, 37.0)	29.0 (21.0, 37.0)	0.920
*Primary outcome*			
Percentage of time with a RASS score of -4 or -5 without rescue sedation, %	100 (96.9, 100)	100 (100, 100)	0.168
*Secondary outcomes*			
Ventilator-free hours within 7 days, h	0.0 (0.0, 34.1)	0.0 (0.0, 3.3)	0.256
Successful extubation	13 (43.3%)	7 (23.3%)	0.100
Length of ICU stay, days	23.5 (8.0, 28.0)	28.0 (8.75, 28.0)	0.841
28-day mortality	11 (39.3%)	17 (60.7%)	0.121
*Adverse events*			
Hypotension	16 (53.3%)	18 (60.0%)	0.602
Hypotension with intervention	5 (16.7%)	7 (23.3%)	0.519
Bradycardia	0 (0.0%)	0 (0.0%)	–

Data are number (%), mean (SD), or median (interquartile range)

ICU: Intensive Care Medicine; RASS: Richmond Agitation and Sedation Scale

At 7 days after enrollment, 35 (58.3%) patients were still in ICU, 13 (21.7%) were discharged from ICU to general ward, 7 (11.7%) were transferred to other hospitals, and 5 (8.3%) died. No significant differences were observed in ventilator-free hours within 7 days, successful extubation, length of ICU stay, or 28-day mortality (Table 2). The proportions of patients requiring mechanical ventilation within 7 days were similar (Additional file 1: Figure S5). Hypotension occurred in 16 (53.3%) patients of remimazolam group and 18 (60.0%) patients of propofol group. No patient experienced bradycardia.

## Discussion

We conducted a pilot study to compare remimazolam besylate with propofol for deep sedation in critically ill patients. We found that the percentage of time within the target sedation range without rescue sedation was similar between the two groups, as well as clinical outcomes and adverse events.

Remimazolam besylate undergoes organ-independent metabolism and is hydrolyzed by tissue esterases into an inactive metabolite. Long-term infusion and high doses are unlikely to cause accumulation or extended effects [5, 6]. The properties make it potentially suitable for deep sedation in ICU patients. Deep sedation or even neuromuscular blocking agents (NMBAs) are required for certain conditions, such as severe ARDS, to facilitate lung and diaphragm-protective ventilation by ameliorating excessive respiratory effort [9–11]. In a multinational study including general ICU patients receiving mechanical ventilation for less than 12 h before enrollment, 50%-60% of patients were deeply sedated for the next 48 h. [12]. Levels of sedation in sedated ICU patients are usually evaluated using subjective scoring systems, such as the RASS and Sedation-Agitation Scale (SAS) [13]. However, when patients receiving NMBAs that cannot communicate or express behavioral reactions, the use of these scales is challenging [14]. In our study, nearly one third of patients received NMBAs. The Narcotrend monitor is an automatic electroencephalogram (EEG) analyzer that provides continuous and objective assessment of sedation [15]. The use of NI to guide deep sedation appears promising.

The median infusion rate of remimazolam besylate was 0.60 mg/kg/h in our study, which was higher than infusion rate in our previous study on patients with light to moderate sedation, in whom was 0.18 mg/kg/h [8]. Hypotension was the most common reported adverse events of remimazolam besylate, but no statistically significant difference was observed. Studies have shown that remimazolam besylate has a better hemodynamic profile than propofol and can be safely used in patients with unstable circulation [16].

Strengths of the present study are that this is the first study comparing remimazolam besylate with propofol in deep sedation, and the level of sedation was assessed using both RASS and the Narcotrend Index. There are several limitations. First, the sample size is small. Second, nurses and physicians were not blinded, as the physical appearance of the two sedatives was obviously different. However, nurses were randomly involved in the care of all the patients during the ICU stay. Third, the duration of study drug infusion was relatively short, and the benefits or risks of using remimazolam besylate for deep sedation beyond 48 h were remain unknown. Fourth, we excluded patients with severe hepatic or renal impairment as recommended by our ethics committee, because the package insert states that limited data are available for these patients.

## Conclusions

In conclusion, remimazolam besylate appears to be an effective and safe agent for short-term deep sedation in critically ill patients. Large sample-sized randomized clinical trials are warranted.

### Supplementary Information

**Additional file 1.** Additional methods and results.

## Data Availability

The datasets used and/or analyzed during the current study are available from the corresponding author on reasonable request.

## Abbreviations

ARDS: Acute Respiratory Distress Syndrome
EEG: Electroencephalogram
ICU: Intensive care unit
IQR: Interquartile range
NI: Narcotrend Index
NMBAs: Neuromuscular Blocking Agents
RASS: Richmond Agitation and Sedation Scale
SAS: Sedation-Agitation Scale
