# Supplementary material for: Remimazolam besylate versus propofol for deep sedation in critically ill patients: a randomized pilot study
Source: Crit Care. 2023 Dec 4;27:474. doi: 10.1186/s13054-023-04760-8 (PMC10694930; doi:10.1186/s13054-023-04760-8)

**Additional file 1. Additional methods and results.**

Table S1. Exclusion criteria.

| - Body mass index (BMI) < 18 or > 30 kg/m^2^ |
| --- |
| - Acute severe neurological disorder or coma |
| - Systolic blood pressure less than 90 mm Hg after appropriate intravenous volume replacement and vasopressors |
| - Heart rate less than 50 beats/min or second- or third-degree heart block in the absence of a pacemaker |
| - Unstable angina or acute myocardial infarction |
| - Left ventricular ejection fraction less than 30% |
| - Contraindicate or allergic to study drugs |
| - Moribund state or not committed to full support |
| - Acute hepatitis or serious hepatic dysfunction (Child-Pugh class C) |
| - Chronic kidney disease with glomerular filtration rate (GFR) < 30 ml/min/1.73m^2^ |
| - Dialysis of all types |
| - Extracorporeal membrane oxygenation therapy |
| - History of alcoholism or intake of anti-anxiety drugs or hypnotics |
| - Myasthenia gravis |
| - Pregnancy or lactation |

Figure S1. Box and whisker plot (25th, 50th, 75th percentiles along with the min and max) for Richmond Agitation Sedation Scale (RASS) during the intervention period.


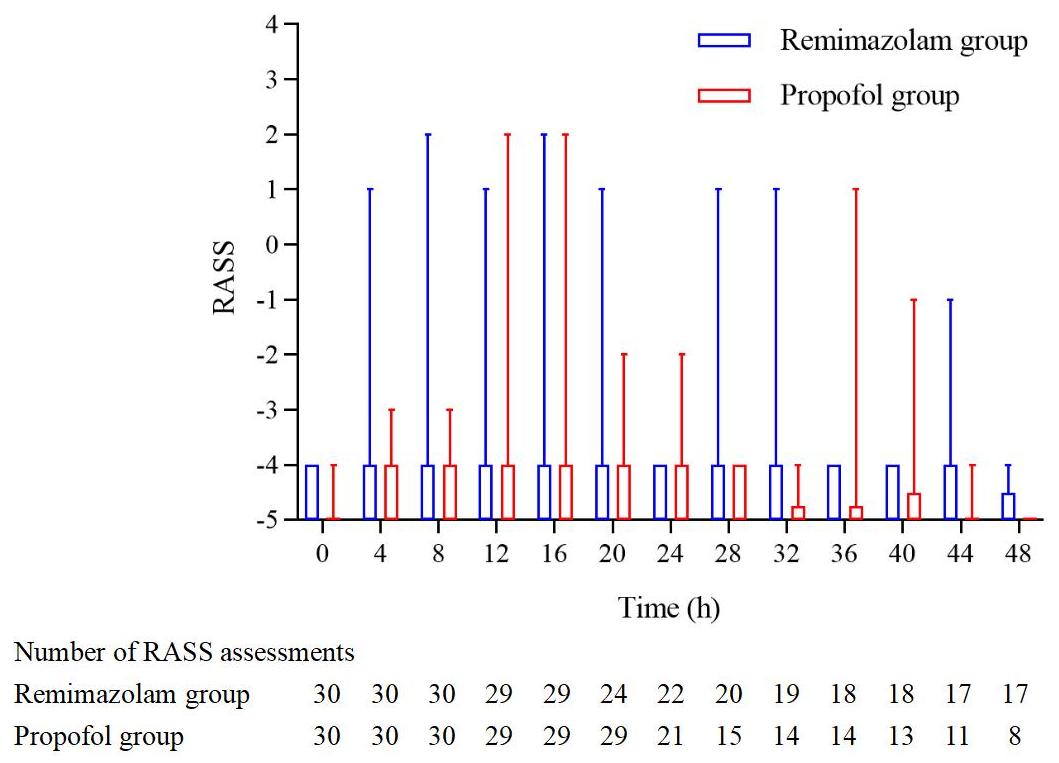


Figure S2. Box and whisker plot (25th, 50th, 75th percentiles along with the min and max) for Narcotrend Index during the intervention period.


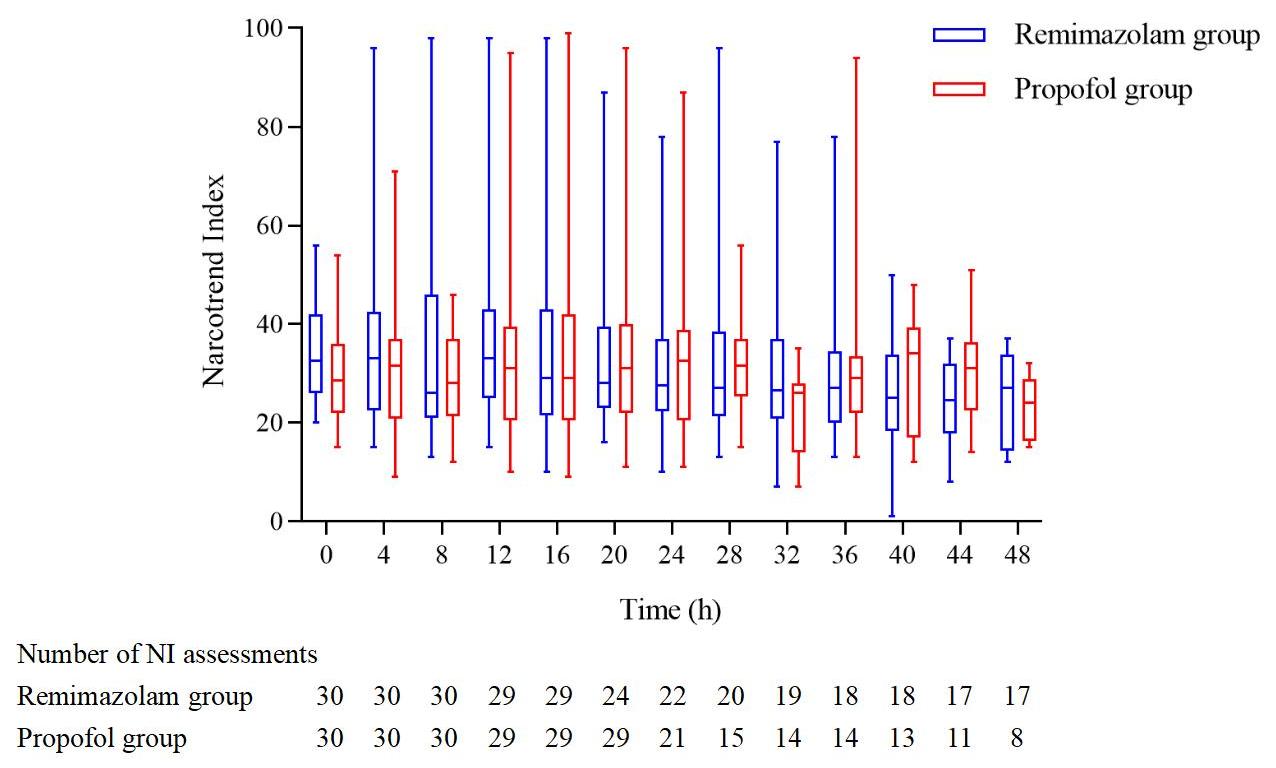


Figure S3. Absolute number of Narcotrend Index assessments.


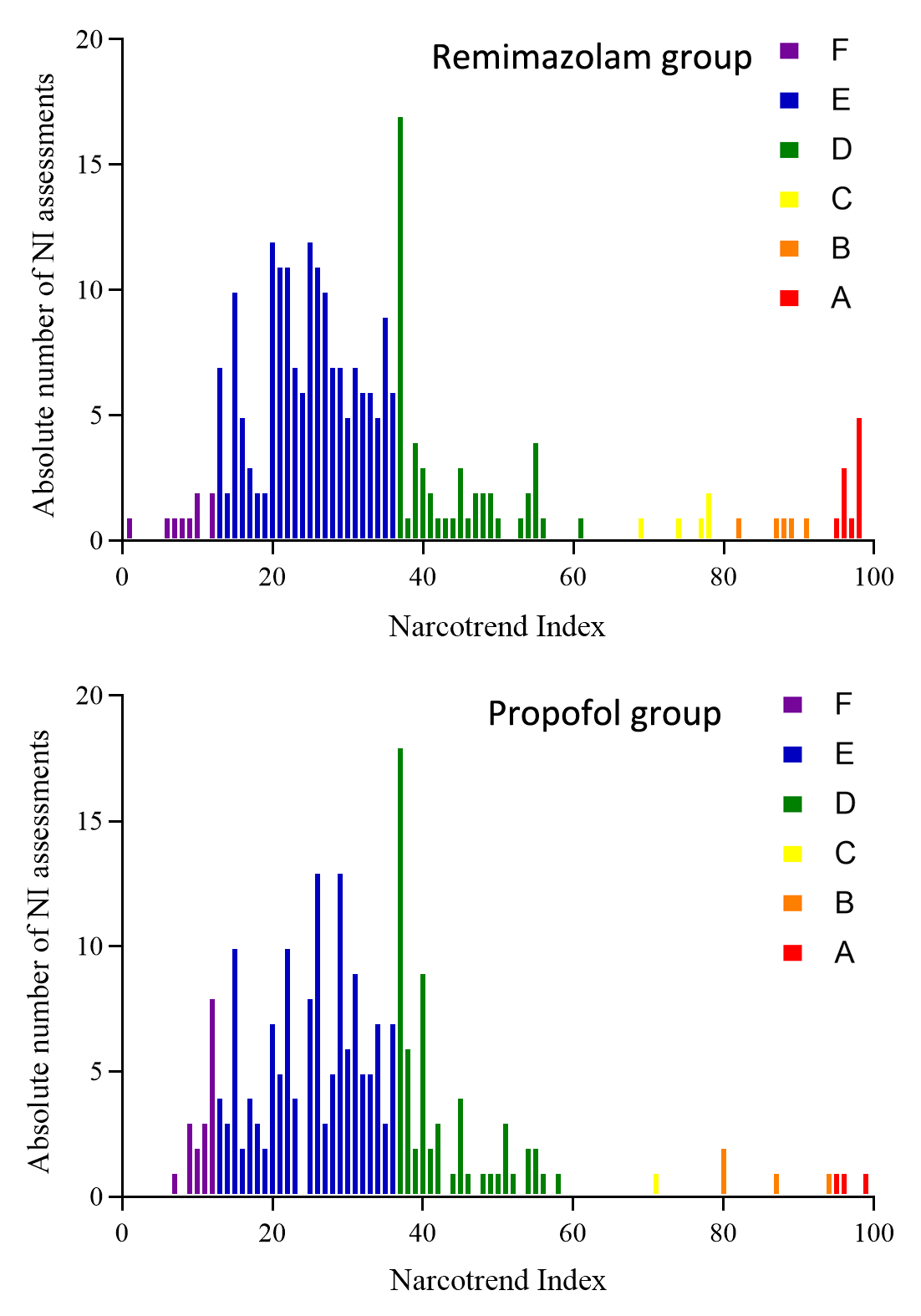


Figure S4. Narcotrend Index values for each RASS scores.


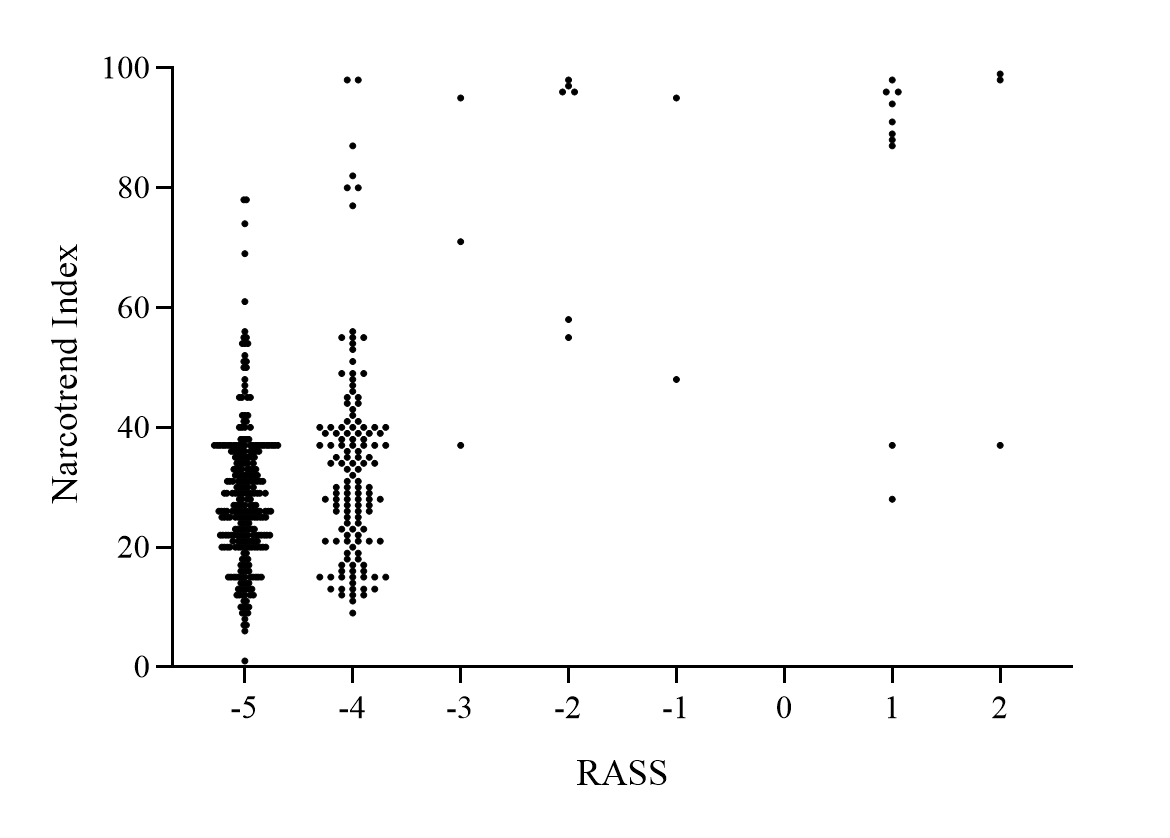


Figure S5. Kaplan-Meier plot of the duration of mechanical ventilation from enrollment to 7 days (Log-rank P=0.21).


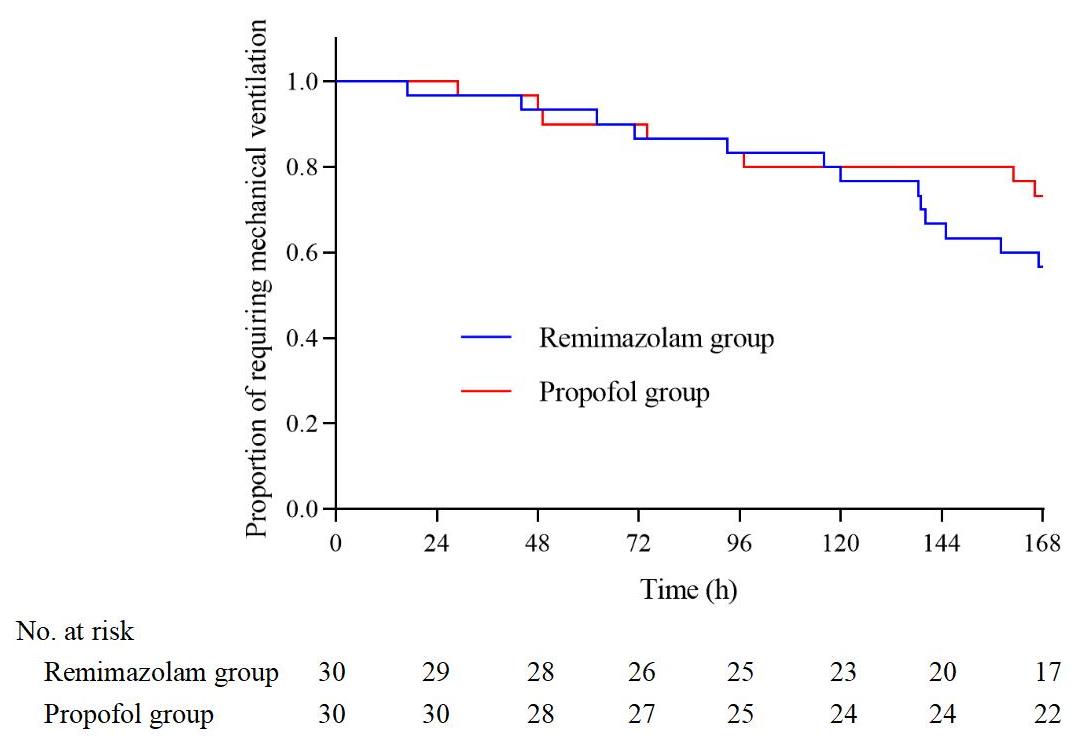

Supplement: Supplementary file 1 — Additional file 1. Additional methods and results. [file 13054_2023_4760_MOESM1_ESM.docx]
